# Addressing Smoking in Supported Residential Facilities for People with Severe Mental Illness: Has Any Progress Been Achieved?

**DOI:** 10.3390/ijerph13100996

**Published:** 2016-10-10

**Authors:** Sharon Lawn, Teri Lucas

**Affiliations:** 1 Flinders Human Behaviour and Health Research Unit, Flinders University, P.O. Box 2100, Adelaide, South Australia 5001, Australia; 2 Cancer Council SA, P.O. Box 929, Unley, South Australia 5061, Australia; tlucas@cancersa.org.au

**Keywords:** smoking cessation, mental illness, hostels, homelessness, ethnography, supported residential facilities

## Abstract

Background: Smoking rates for people with severe mental illness have remained high despite significant declines in smoking rates in the general population, particularly for residents of community supported residential facilities (SRFs) where smoking has been largely neglected and institutionalized. Methods: Two studies undertaken 10 years apart (2000 and 2010) with SRFs in Adelaide, Australia looked at historical trends to determine whether any progress has been made to address smoking for this population. The first study was ethnographic and involved narrative description and analysis of the social milieu of smoking following multiple observations of smoking behaviours in two SRFs. The second study involved an eight-week smoking cessation group program providing tailored support and free nicotine replacement therapy to residents across six SRFs. Changes in smoking behaviours were measured using pre and post surveys with residents, with outcomes verified by also seeking SRF staff and smoking cessation group facilitator qualitative feedback and reflection on their observations of residents and the setting. Results: The culture of smoking in mental health SRFs is a complex part of the social milieu of these settings. There appears to have been little change in smoking behaviours of residents and attitudes and support responses by staff of SRFs since 2000 despite smoking rates declining in the general community. Tailored smoking cessation group programs for this population were well received and did help SRF residents to quit or cut down their smoking. They did challenge staff negative attitudes to residents’ capacity to smoke less or quit. Conclusions: A more systematic approach that addresses SRF regulations, smoke-free policies, staff attitudes and training, and consistent smoking cessation support to residents is needed.

## 1. Introduction

In Australia, smoking rates for people with severe mental illness (SMI) (such as schizophrenia, bipolar disorder, and major depression) are high and have remained unchanged for more than a decade, despite their significant decline in the general population and significant public health efforts [1,2]. The second Australian national survey of people with a psychotic illness found that 68.9% of this population smoked in 1997–1998 compared with 67.2% in 2010 [3]. Smoking rates for the general population for the same period fell from 26% to 20%, with current rates at approximately 15.9% [2]. People with SMI usually start smoking at an earlier age, smoke more cigarettes per day, and are more nicotine dependent compared with the general population [4]. Consequently, their life expectancy is reduced by 20 years, largely due to tobacco-related disease [5,6]. The cost of smoking by people with mental illness in Australia has been estimated to be more than $30 billion per year [7]. Much of the existing research in Australia and internationally about smoke-free policy and mental health has focused on in-patient settings [8,9,10]. However, most people who require mental health care in Australia receive their care within the community where they reside in their own homes, public or private rental accommodation, or supported residential facilities (SRFs) [11]. In Australia, approximately 20% of adults experience a mental illness each year [12]. The rate of mental illness among SRF residents in Australia is unknown, though it is likely to be very high given their complex psychosocial circumstances. Of the total population of Australians with SMI, the proportion that live in their own homes, are inpatients, or that are living in SRFs is unknown. However, approximately 3% of Australians are estimated to experience psychosocial disability caused by the effects of mental illness [12], suggesting that approximately 1 in 50 South Australians with SMI reside in an SRF.

The term “SRF” is used widely in Australia as an umbrella term for low-level supported accommodation made available to people who are unable to live independently and who have limited financial means other than a government benefit or pension. A number of terms are used in Australia and in other countries to denote this type of accommodation such as “hostel”, “boarding house”, and “rooming house”. Features in common are that they provide accommodation and care services to older people and people with psychosocial disabilities (18 years and over) in a group setting. Services provided may include personal care, meals, medication monitoring, cleaning, and laundry. SRFs are not aged care facilities; however, they may house individuals over the age of 65. This is because they have been long-term residents of the facility, cannot afford aged care accommodation, or have a disability that precludes them from having their needs managed appropriately in an aged care facility. In Australia, SRFs are regulated by the Supported Residential Facilities Act 1992. They are privately operated and subsidized by the government, with no restriction on who can refer the individual to an SRF [13].

SRFs by their nature provide a home for people with significant psychosocial disability. Many of these individuals would otherwise be homeless or reside in long-term inpatient psychiatric care settings [12]. Many people residing in SRFs have a diagnosed SMI and are currently receiving or have previously received regular mental health support and treatment from a primary care or community mental health service [13,14,15]. Similar high rates of mental illness have been noted in emergency accommodation that serves the needs of the homeless in Australia [16]. The impact of the SRF environment on overall health and wellbeing has not been widely researched. Research on aged care facilities providing care to residents with cognitive issues provides some sense of the potential range of impacts of institutionalization on SRF residents. For example, larger sized units have been shown to be associated with greater levels of agitation, intellectual deterioration, emotional disturbances, territorial conflicts, and aggression towards others; and smaller units are associated with higher rates of depression and anxiety [17,18,19,20]. A systematic review comparing housing schemes for those with SMI found the pervasive outcome for most residents was a risk of increasing dependence on professionals and prolonging exclusion from the community [21].

Currently in Australia, partial smoke-free policy applies to SRFs. They are excluded from total smoke-free policy legislation afforded to other shared public spaces because they are deemed as being “residents” homes. Therefore, policy and practice must navigate across SRFs being both public and private spaces. In virtually all states and territories in Australia smoking is not permitted in public areas within SRFs such as common rooms, dining rooms, and kitchens. However, residents are able to smoke in designated areas, although there is great variability between jurisdictions [2]. The South Australian Supported Residential Facilities Act 1992 [22] makes no mention of smoking or other drug use by SRF residents, though it does include a number of principles to be observed in the management and administration of SRFs. These principles include high quality care, a home-like environment, a safe physical environment, privacy, dignity, and respect, “the right to participate in activities of their choosing (so long as they do not unreasonably infringe upon the rights of others) and must not be subjected to exploitation of their financial or other assets” (p. 6). The implementation of these principles into care within the SRF environment in relation to the role and influence of smoking has not been investigated.

In South Australia in 2002, there were approximately 65 SRFs across the state, within a total state population of 1.5 million [23], housing between 1300 and 1500 SRF residents. Facilities ranged in size from 4 to 64 residents [13]. In 2012, there were 29 pension-only SRFs in South Australia (a total of 1012 licensed beds) within a total state population of approximately 1.65 million [24]. In most cases, the only form of income or assets among SRF residents was a government pension or benefit and rent assistance with approximately 80% or more of their total income paid to the SRF [14] (p. 17). Approximately 60% of SRF residents were aged between 41–64 years, with 20% aged 65 years and over, and 20% below 41 years of age. The limited financial situation of SRF residents means that they have little money available to purchase personal care items beyond money spent paying for their accommodation. No research has been conducted to determine the economic and social impacts of smoking on their quality of life.

The limited research that exists on the general psychosocial needs and experiences of residents of SRFs and the staff who provide care to them offers some context for understanding the situation for SRF residents who smoke. Taylor et al., in their systematic review of institutional care for people with longer term mental health needs, made a number of recommendations for how mental health community-based care institutions should look and how they should operate. However, their focus was on clinical care rather than SRF non-clinical care environments where staff are largely an unskilled workforce [25]. In the U.S., Baker and Douglas undertook a large study to investigate outcomes for people with mental health issues living in supported and unsupported community housing [26]. They found that supported housing residents living in poor quality living environments had significant maladaptive behaviours such as high rates of smoking and other drug use. An Australian qualitative study involving interviews with 14 boarding house residents [12] found that, although residents regarded staff favorably, they experienced significant threats to their quality of life and hope for their future due to aspects of daily living and relationships with others within the boarding house environment. Smoking was not mentioned directly in that study. Another Australian study involving 60 people with schizophrenia residing in hostels/boarding houses [27] (p. 323) found that they regarded this accommodation as “asylum or sanctuary from the outside world”. A systematic review of 28 studies of supported housing for people with mental health problems [28] found that the poor quality of the physical environment and the limited degree of privacy were strong mediators of residents’ poor psychosocial health outcomes. Again, the role of smoking was not directly investigated in that review.

In summary, there has also been very limited research and documentation of tobacco use in the SRF environment internationally [29]. The use of cigarettes as a form of currency, as a social “connector”, to relieve boredom, and as a means of the managing behaviour of patients by staff in inpatient psychiatric settings has been described in detail [30]. SRFs are likely to mirror these cultural patterns because of the nature of these settings and the populations they serve [12]. Smoking status amongst residents in most South Australian SRFs is currently not recorded as a standard component of data collection and so no accurate data is available on the percentage of residents who use tobacco. However, anecdotally, smoking rates are perceived to be about 70%–80% at many sites [13]. These rates are very high when measured against the wider community daily smoking rates of 15.7% for South Australians aged 15 years and over in 2014. We know that smoking rates for the broader population of people with SMI have remained relatively unchanged despite a range of assertive public health and policy measures [1]. This is likely to also be so for people with mental illness residing in SRFs who are among the most disabled in the SMI community.

An extensive search of the Internet and research databases (PubMed and PsycINFO) found no studies that directly investigated smoking, smoke-free policy or smoking cessation support interventions for people with SMI in community SRFs. We therefore drew on the context of the related areas of homelessness and institutional care settings, given that many residents of SRFs are perceived to have similar levels of need, disability, and psychosocial experiences. Several studies have investigated smoking cessation support for homeless people [31,32,33,34,35,36]. In a recent New South Wales study with 144 male smokers who were homeless, for example, 66.2% reported having a diagnosed mental illness and three-quarters of these reported smoking to alleviate symptoms of their mental illness [16]. Chen et al. undertook an anonymous voluntary survey with 100 smoking individuals residing at a homeless shelter, assessing high-risk smoking behaviours and respondents’ perceived barriers to long-term smoking cessation [37]. They found 90% reported engaging in at least one high-risk tobacco practice. Nicotine replacement therapy (NRT) was the most desired form of smoking cessation aid, with use of tobacco smoking to alleviate stress and anxiety reported as the most significant perceived barrier to smoking cessation. They reported that high-risk tobacco practices are very common among this population and stressed the need for further research in this largely neglected area. 

Recent evidence indicates that treating tobacco use by people with mental illness is effective and desired, even by those with SMI [38,39,40]. There is no evidence to suggest that people with SMI residing in SRFs might be less motivated to smoke less or quit. Yet it is unclear whether residents of SRFs are offered smoking cessation advice and treatments and whether such interventions are successful in this population and setting. Social researchers and mental health advocates have expressed significant concerns that many SRFs are little more than asylums in the community [41,42,43]. For example, in the launch of his seminal report to the national government on the human rights of people with mental illness Burdekin stated, “It is also appalling that homeless shelters, refuges and boarding houses are now functioning, de facto, as a major component of the ‘accommodation’ provided by our society for thousands of Australians affected by mental illness. The living conditions in many of these establishments are disgraceful. Few have trained mental health workers on staff, and there are rarely any decent opportunities for rehabilitation” [43].

The current study aimed to “shine a light” on the issue of smoking in SRFs in order to promote smoking cessation efforts for this population. To do this, the study aimed to help fill a gap in the existing literature by comparing the findings of two studies [29,44,45] on smoking in the SRF setting, conducted approximately 10 years apart (2000 and 2010), to determine whether any progress has been made to address smoking for this population and to discuss the factors that may be influencing this situation. In summary, the purpose of the paper is to draw conclusions about historical trends with regard to resident and staff attitudes about smoking, smoking cessation, and environmental factors related to smoking and smoking cessation in SRFs in Adelaide, Australia.

## 2. Experimental Section—Design

This paper reports on two studies involving smoking in SRFs conducted in Adelaide, South Australia, The study conducted in 2000 by the first author is described first and involved participant observation of two SRFs and narrative description of these settings. The first study was part of the first researcher’s original PhD thesis on the culture of smoking across mental health settings in Adelaide. The second study conducted in 2010 by the second author is then described. It involved the delivery of tailored smoking cessation support groups to residents in six SRFs. The second study was informed by the results of the first study and prompted by a commitment by the Cancer Council of South Australia (known as Cancer Council SA) to investigate how it might support smoking cessation in South Australia’s SRF sector as part of its broader “Tackling Tobacco” agenda.

## 3. Experimental Section—The First Study—2000

### 3.1. Methods

In the first study conducted in 2000, participant observation of the setting was undertaken to produce a narrative description and analysis of the social milieu of smoking in the SRF setting. Given the lack of existing research in this field, participant observation was useful to explore and provide a rich description of the role of smoking in the SRF setting [30,44,46]. Participant observation is a useful way of researching a culture from within [47]; “for studying processes, relationships among people and events, the organization of people and events, continuities over time, and patterns as well as the immediate socio-cultural contexts in which human existence unfolds” [48] (p. 12). It is particularly useful when little is known about a phenomenon, when differences exist between the view of insiders as opposed to outsiders, when a phenomenon is obscured from the view of outsiders, when it involves taboo issues, or where the sensitivities and cognitive difficulties of many people in this setting would make other methods such as interviews difficult [48]. Direct contact with the environment also allows greater understanding of the context and therefore provides a more holistic approach to the interpretation of the data [49]. All of these conditions were thought to exist in the community mental health SRF setting.

#### 3.1.1. The Pilot Period

A one-month pilot study preceding the main participant observation period was performed by the first author to gain familiarity and rapport with the participants (residents and staff). This habituation improved validity and reliability of observations by allowing the observed residents and staff to become familiar with the researcher’s presence and go about their usual day in their usual manner without staging activities or behaviours for the researcher. The pilot period was also used to identify and resolve any problems and barriers to access, to test observation sheets that had been devised to help record interactions and observations, and to make any adjustments accordingly. Regular meetings with an independent auditor to enhance validity and reliability of the study processes occurred throughout the total participant observation period.

#### 3.1.2. The Setting

Participant observation occurred over a period of six months in the second half of 2000 and early 2001. Two community SRFs within the mental health service catchment area were selected, based on them being representative of SRFs in their region. SRF 1 was known to provide accommodation for older and younger residents (ranging in their 20s to 90s). SRF 2 accommodated residents who were generally younger (under 50 years). Each SRF accommodated 24 residents, with several in shared rooms with between 2–4 other residents. They were approximately 12 km apart and owned and run independently of each other by private providers. Each SRF was visited multiple times at different times during the day and different days of the week to ensure capture of a broad range of conditions. All observations took place in the main outdoors covered seating area within the grounds of each SRF. These areas were not officially designated smoking areas; however, they appeared to be used extensively for that purpose by residents and staff.

#### 3.1.3. Observation Sheets

Observation sheets were used, based on themes that emerged from qualitative interviews with clients and staff of mental health services during the earlier phase of the project reported elsewhere [30,44,46]. Observation sheets served a range of purposes: to record the level of smoking; to record smoking behaviours and interactions between those participants present in the observed settings; and to note the social and environmental context within which smoking occurred in order to describe the social milieu of smoking. Ideas for the structure of the observation sheets were gained from adaptation of observation instruments used for evaluation of classroom interactions [50]. Observed behaviours and interactions using sequential and frequency observation sheets were counted and recorded using ticks (Appendix Sheet A1, Sheet A2 and Sheet A3).

Extensive descriptive reflective notes were also kept to illustrate the meaning and impact of what the researcher observed, including the researcher’s own feelings and reactions to observations. All observations were noted as they occurred or as soon after as possible, with detailed recording of date, place, time, main activity, and those present during observations [48]. Details of the observation sheets are included in Box 1 and as supplementary material.

Box 1 **Observation sheets used in the study.**

(1) Unobtrusive measures—measures of physical aspects of the environment such as wear and tear in specific areas and use of ashtrays, as noted by Patton’s reference to the study by Wolf and Tymitz measuring wear on rugs to indicate popularity of particular areas of the National Museum of Natural History Smithsonian Institute [49].

(2) Sequential measures of interactions between participants—looking at roles, types of behaviours and responses, source, direction of behaviours, modes of communication and their functions and the circumstances in which certain behaviours occurred.

(3) Descriptive measures—detailing the type of encounters and social milieu of the settings in which they were observed, the composition of the group, and their language and behaviours.

(4) Frequency measures—counting of particular types of events and interactions over set periods of time as a proportion of time spent on overall roles for staff and overall activity for residents. This included counting the consumption of cigarettes for participants over set 10-min intervals because this was the average length of time that the researcher observed that it took to smoke a cigarette. Measures also reflected the proportion of people engaged in certain activities and the proportion of time they spent in those activities.

(5) Dummy observation—giving the appearance of actively observing and recording at times when this was not the case. Likewise, appearing not to be observing and recording when this was in fact the case. This improved validity and reliability of observations, to avoid “staged” activity by participants.

#### 3.1.4. Ethics

Ethical approval was granted by the Flinders Clinical Research Ethics Committee (166/98) and the Royal Adelaide Hospital Research Ethics Committee (000214). Permission was gained from the SRF manager of each setting. This involved providing each SRF manager with a formal letter and organizing a face-to-face meeting with them to detail the aims of the research and proposed procedures. The first author also sought and gained the permission and advice from two mental health consumer advocacy groups within the region prior to entering the field. This was particularly important because the participant observation methods meant that distribution of participant information sheets and seeking SRF participants’ signed consent prior to data collection was impractical and their informed consent could not be universally assured [51,52]. All data collected was de-identified. No inducements were provided to any participants. 

### 3.2. Data Collection

Two community SRFs were visited six times each (12 visits in total) to record data in the area immediately outside the entrance door to the SRF that was used by residents for smoking and daytime outside seating and recreation. Visits to the first SRF occurred between the hours of 6.30 am and 9.30 pm over seven days. Visits to the second SRF occurred between the hours of 7.30 am and 10 pm over seven days. The researcher was present in each setting for varying lengths of time as observer and participant, dependent on the setting, the needs of SRF staff and residents and the circumstances on the day. Following negotiation of entry, settings were visited at random with no predetermined order in mind. Visits occurred in blocks of three to five hours, with sequential and frequency data collected during continuous three-hour blocks whilst at each site. Number of visits to each setting was determined as the participant observation proceeded. Decisions to perform further visits were made on the basis of any patterns emerging from the observations. Decisions to cease further visits were made once repetition of patterns of behaviour and observation of environmental aspects occurred at least four times. For example, at the first visit a series of general observations were made. These observations were either observed with enhanced understanding or refuted at the second visit. The third visit was an opportunity to confirm the presence of particular aspects observed in earlier visits, and further visits were done to verify previous observations. Where unexpected observations were made, these were noted and hypotheses were made and tested by further observations, reflection, and discussion with participants, the auditor, and study supervisors. The researcher was diligent in keeping a reflective journal throughout the process of data collection.

### 3.3. Data Analysis

Descriptive statistics were used to analyze and report results of frequency measures of smoking consumption, number of people in the observed environment, extent of passive smoking and smoking behaviours collected in observation sheet data. Inferential statistical analysis was also undertaken to test and report on any comparisons and patterns across the types of settings involved in the original larger study. These are reported elsewhere [29]. Qualitative analysis of unobtrusive, sequential, and descriptive data was undertaken as a narrative account—“A Day in the Life of…” to capture the essence of observations [53,54]. This data collection and data analysis occurred simultaneously using the constant comparative method of checking and cross-referencing the observation data and reflections. Journal notes, memos, supervision notes, and artefacts were organized and arranged according to each setting. They were then chronologically ordered so that all the data pertaining to each setting could be read and re-read several times and coded for recurrent themes and patterns, and any leads followed up. At that time, the first author’s PhD supervisor (R Pols) acted as second coder and discussant of the data. 

## 4. Experimental Section—The Second Study 2010

In 2010, the Cancer Council SA conducted a pilot program called “Tackling Tobacco in SRFs” in collaboration with the SRF sector. This pilot program was prompted by growing concerns expressed by service providers across the mental health and tobacco control community about the observed high rates of smoking in South Australia’s SRFs. They also expressed concern that the needs of this population were being neglected whilst smoking cessation efforts were progressing in other areas such as public places, hospitals, workplaces, prisons, and in the community more broadly. Prior to the pilot program no SRF-specific, formalized initiatives were known to exist in South Australia to encourage or support SRF residents to attempt to reduce or quit smoking.

Informed by the findings of the first study and a decision by the Cancer Council SA in 2009 to investigate the smoking situation within South Australia’s SRFs, the second researcher commenced the “Tackling Tobacco in SRFs” project. This involved preliminary visits to SRF sites across Adelaide to scope their interest in being involved in the project. Anecdotal evidence gained from the researcher’s discussions with SRF staff and residents during that time was that the attitudes and behaviours of both staff and residents (and family members, where they were in contact with the resident) reflected smoking as entrenched and normalized within SRFs. Preliminary comments from SRF staff to the researcher included: “Don’t bother, they all smoke”; “They are hopeless, they can’t quit”; and “It’s just what they do, they love it”. Similarly, anecdotal comments from residents reflected how smoking was an entrenched activity in their lives: “No, I can’t quit”; “I just love smoking”; “There’s nothing else to do”; “I know it’s bad for me but it’s hard to give it up”; and “It’s too hard for me to quit while I am here (living in an SRF)”. Comments from residents’ family and friends during these preliminary discussions showed that they perceived smoking as a “small luxury” for residents “when they are deprived of so much else in life”. Some SRF staff voiced reluctance to support smoke-free policy in the SRF setting, perceiving smoking as an integral part of life in SRFs. 

This pilot program was a joint project initiated by the Cancer Council SA and undertaken with the active support of the SRF Health Assessment Team (HAT), a government-funded authority that worked across the SRF sector. A steering committee was formed in 2009, comprising representatives from Quit SA, the SRF HAT team, and primary health community services. It also involved mental health clinicians and mental health peer workers from the Tobacco and Mental Illness Program that had been established several years earlier to provide dedicated community smoking cessation support groups and printed resource materials to people with mental illness across the mental health service sector in South Australia [55]. A framework was drafted for the project activities, the first smoking cessation support group sessions were scheduled for January 2010 and SRFs across metropolitan Adelaide were invited to participate in the program. 

### Methods

Invitations were sent out to SRF managers across metropolitan Adelaide, and six SRF sites expressed interest in participating in the pilot project. These sites were distributed across the metropolitan area (three southern, one central, and two northern region). The researcher and staff from the various organizations involved in the project facilitated smoking cessation groups in each location. The researcher visited each SRF to undertake an environmental scan prior to commencement of the weekly group sessions. Advice, posters, signage, and support for staff and managers were given to each SRF site. These visits also served as an introduction to the SRF residents in preparation for the pending visitors and group programs and their potential participation. Where possible, designated smoking areas were created within the SRF facility for residents to use if these areas did not exist. 

#### The Smoking Cessation Support Groups

A series of eight structured group sessions were provided at each SRF. The sessions were designed to create rapport with participants, to ascertain their individual knowledge about the impact of smoking, to educate and inform them about the use of NRT, and to provide support to participants to reduce their smoking or quit. There was provision for flexibility in each session to address specific questions or concerns expressed by participants, and to accommodate any differences in the type and level of support provided at each site. Each region had differing levels of support to conduct the group program. The researcher and a community nurse facilitated the group sessions at the two southern sites, two staff from the primary health care team facilitated groups at the central and one northern site, and the group at the other northern site was facilitated by the researcher. Each group session ran for approximately one hour and was scheduled for mid-morning. SRF managers advised that this time would be when most residents would be awake, most alert, and less likely to have other commitments. Participation in the group program was voluntary and participants could arrive and leave the group as they wished or needed. 

Handouts that had been developed for the Tobacco and Mental Illness Program were piloted during the group sessions. Some handouts were adapted to satisfy requests from participants (for example, how to cope with boredom within the SRF context) and some were modified in order to accommodate the needs of residents with higher levels of psychosocial disability. A commitment to quitting smoking was not a pre-requisite for their attendance. SRF staff offered encouragement and support for residents’ attendance and could also join the group sessions if they wished. Medical clearance was sought from the residents’ GP prior to the provision of free NRT products. The weekly sessions aimed to:

- Inform residents and staff about the pilot program;
- Educate residents and staff about the hazards of smoking, including the direct physical consequences of active and passive smoking, and the risks around cigarette lighters and other sources of ignition;
- Engage and support staff to quit if they chose to attempt smoking cessation;
- Up-skill SRF staff to provide cessation support to residents;
- Prepare residents for the challenge should they decide to attempt to cut down or quit;
- Provide education and distribute the free NRT offered as a part of the program;
- Engage residents with new strategies for quitting;
- Encourage and support a focus on living a healthy life by not smoking, eating well, having a moderate intake of alcohol, and getting some physical activity on a daily basis; and
- Mark progress and celebrate achievements.

#### Data Collection Measures and Their Analysis

Brief pre and post surveys were developed to collect information from SRF residents about any impact of the group program on their smoking behaviour. Each participant completed these surveys independently, or with the assistance of the group facilitator, if requested. The pre survey asked if they were a smoker, how much they smoked, their interest in quitting or cutting down, and their preference for using NRT. It also asked them about their level of nicotine dependence using the brief Fagerstrom Scale (How soon after waking do you smoke your first cigarette? How many cigarettes to you smoke each day?) [56]. The post survey asked if they had tried to quit smoking during the program, whether they were currently smoking or quit, and what NRT they had tried and preferred. Because several participants had significant psychosocial disability, survey questions and processes were deliberately brief to minimize burden. 

To enhance rigor, the researcher also sought verbal feedback from SRF staff at the conclusion of the group programs in order to validate group participants’ comments and to gain staff impressions of the impact of the group programs. The process of seeking SRF staff feedback involved the researcher visiting each site and speaking with those individuals who wished to provide comments. They asked SRF staff for feedback on what they observed in the SRF environment and residents’ smoking behaviours. For example, where a participant had reported a reduction in their smoking, the SRF staff reported their observation of this and reduced distribution of daily cigarettes to that resident. 

The researcher also gathered process feedback from other group facilitators as part of regular communication, periodic co-facilitation of group sessions at each site and support provided throughout the project period. This also included any comments about smoking and the smoking cessation group made by SRF residents and staff that the group facilitator had noted from their contact with the SRFs. The researcher also kept a reflective diary throughout the project period, capturing the above communications and their own impressions of what they saw, heard, and felt in order to inform their final report on the project. This included the formulation of case study examples from sites and individuals. Group facilitators kept records of how many residents attended each session, noting their progress, their use of NRT, and any issues of concern expressed by residents. 

## 5. Results

The results of the first study conducted in 2000 are provided first, followed by the results of the second study conducted in 2010. Then, a brief discussion of the study results together in terms of themes is provided in order to integrate the key results.

### 5.1. Results for the First Study—2000

At the conclusion of data collection, the audit report confirmed that the research was conducted ethically according to clearly described and justified methods and that a clear audit trail was established [57,58,59,60]. 

Details of the number and type of observations are given in Table 1. This is followed by a brief description of the main features observed in the SRFs. A narrative account of “A Day in the Life of…” is then provided to capture the observed scene of typical interactions involving smoking in each SRF [29] (see Box 2). All data is de-identified.

The residents of SRF 1 were predominantly in older age groups (50 years and over) but there were some younger residents (under 50 years). Its environment was highly regimented by staff, with several residents required to ask staff for their cigarette ration for the day. SRF 2 housed residents who were predominantly under 50 years of age. Many of these residents were away from the SRF during the day and returned for the evening meal. Of note was the higher number of cigarettes consumed compared to the number of smokers during the observations that occurred in the early morning at each site. The role and intensity of nicotine dependence and withdrawal among residents may have played a role here. Some residents were given their morning ration of three to five cigarettes at the start of the day and chose to smoke them as quickly as they could. This meant that they had no cigarettes for several hours until they received their afternoon ration after lunchtime. The consistent and repeated use of the smoking areas by several residents throughout the day was noted, as was the high level of staff smoking interaction in the smoking area of the second SRF. The frequency and high number of passive smokers was also noted, with several non-smoking residents observed sitting next to smokers in order to inhale their cigarette smoke, presumably when they had run out of their own supply. Differences between each SRF were noted, with the number of cigarettes consumed and the overall proportion of smokers being higher at the second SRF. This may be due to the resident population at this site being generally younger and more able to move in and out of the area. In the first SRF, many residents were frail and spent much of their day sitting indoors in the shared lounge area, in their beds, or in the smoking area.

Box 2 **“A Day in the Life of…” Observations from the Community Hostels (2000).**

***The First Community Hostel: (beds = 24)***

Break of day at the hostel. It’s 7.30 am. It’s cool and mild. The smoking area is deserted. The chairs are lined up ready for today’s activity. The seven ashtrays have been emptied the previous night and are lined up ready for today’s deposits, though already they have received the offerings from those who couldn’t sleep. The ten kilogram coffee tin, now a makeshift ashtray, sits amongst them ready to help cope with the load of butts envisaged for the day. The concrete has been swept early; the table sparkles. From inside come the sounds from the kitchen as the cook busily prepares breakfast. An occasional sound of showers and running water as the clients slowly emerge from their beds.

A middle-aged man comes out in his pyjamas, half down on one side, and surveys the smokers’ table and sifts through the ashtrays for butts. No luck yet today; they’re all smoked down to the filters; straight back inside. The usual three elderly ladies come out together. They light up their smokes and make small talk. “I like your jumper”. “It’s Tuesday today, isn’t it?” The middle-aged man rushes back out now, stands over the ladies and immediately asks them for a smoke. The second lady says, “I’m not meant to give them to you”, but she relents. He makes conversation with them as they all smoke together. Two male clients come out and take up positions alongside the ladies.

The staff member comes out. “You didn’t give him one did you? He’s not meant to ask for them. I’ll get his in a minute. You mustn’t give them away. He gets his own money for smokes”. The tall lady replies, “No, we didn’t give him one”, as they all look like the cat that swallowed the bird. The staff member shakes her head and returns a cigarette to her to make up for the one she gave away.

When the staff member is gone, they all confer and stress to the second lady that she only gets five cigarettes and they have to last her for the morning. She immediately gives this cigarette to the man. “Now you only have four left”. The three women leave and the three men stay. The middle-aged man breaks wind loudly as he smokes his second cigarette. He stands pressured, restless, already lining up his next cigarette as he finishes his second. He persists with one man and then the other, over and over again asking, “You got a smoke? You got a smoke?” They remain silent. He returns inside to try his luck later.

The staff member moves busily here and there, getting breakfast organized and supervising the showering, as she is the only one on duty so far. One lady has been incontinent again. A mop and bucket appear instantly. The smell of cigarettes and toast and the sound of residents being directed here and there fill the morning air. The three ladies have returned to have a smoke. A small lady has sat down and begins patiently to roll a cigarette. The middle-aged man is outside again. He stands silently over her, insistently. He has apparently cornered her inside. The coughing is horrendous from all. She tells him to sit but he says no, preferring to stand, shuffling his feet in expectation. She hands him a smoke and proceeds to roll herself one. Through the sliding door, a lady in the adjoining room sits patiently as the staff member attends to her personal care. The staff member hands her the three cigarettes she is rationed for the morning. Her eyes beam as she holds them tightly between her fingers. “OK sweetheart, enjoy”. The middle-aged man has, in the space of thirty minutes, managed to bott (cadge) five cigarettes so far. He persists again as he stands over one lady, holding a half-smoked cigarette behind his back and asking for another one. She yells back at him after his third plea, “No.” He eventually leaves. Before she finishes her first cigarette, she lights the second from it. The third follows in similar style. No more now till late afternoon, unless someone responds to her pleas beforehand.

It’s 9 am now. Another lady is showered, dressed and she’s off to the bank and then local shops to buy more cigarettes. She returns in minimum time. She says she plans to sit and smoke for most of the day. “I’m sixty soon. I’ve done my jobs for the day, so I can do what I want to now”. A younger man in his late twenties sits quietly at one end of the table, in the corner as he does most days, smoking, silently observing the antics of the middle aged man and the others. He’s passing time. A young man in his early twenties quickly comes out to the smokers’ area. “Do you want some coke for a smoke; please, do you want some?” “No”, the elderly lady replies gruffly. He immediately moves to the next person and gets the same response. He goes to the butts and lights what seems like nothing left of a cigarette and sucks quickly on it. He persists with the ladies. “It’s nice and cold. You can have as much as you want? Please?” he asks each person who comes out. There are nine smokers now. The elderly lady who had given one away previously says, “I’ll get into trouble if I give you one”. He leaves abruptly again. Another elderly lady returns from her brief walk, through the cloud of smokers. She’s an ex-smoker and rarely sits in this area now due to her asthma. She huffs and puffs as she goes. The ladies congregate again in peaceful, polite conversation. They share a light and smoke, attempting the small social graces. The young man comes again, holding out his coke. This time, he’s successful with the lady who rolled her own beforehand. They all sit or stand around smoking, concentrating, saying little to one another.

***The Second Community Hostel: (Beds = 24)***

Mid-morning at the hostel, coming up the driveway, the area is strewn with residents sitting on the plastic seats or on the curbing, or standing, pacing aimlessly and all smoking. Most are in their twenties and thirties. They have many more years of this existence. I have the overwhelming sense of this not being a home, rather a place for people to exist in parallel, together but separate, as few words are exchanged with each other, only the occasional vacant look or stare. A young female resident tells me, “It’s really hard here because everyone smokes. I find I’ve got less to look forward to the longer I’m here. I spend most of the day doing the circuit of the hostel, pacing through the building and up and down the drive, over and over, smoking”. This is a common activity for many residents.

Late afternoon now and we’re sitting under the large veranda at one of the many tables and chairs. Ashtrays are strewn throughout the area although the blackened concrete, strewn with cigarette butts, suggests that few people use them. Twelve residents sit or stand as a fragmented group; few talk to each other. Next to me, one man looks beseechingly and attempts the social graces. He deserves whatever conversation I can offer him. He talks about his dreams of meeting a lady and having a life together. He’s been here for five years now. He manages a half smile through his blackened, crumbling teeth. His fingers are stained from many years of smoking. Life here appears to have a dampened pace. “It’s just good to have someone to talk to. You know you can get pretty lonely, even when there are other people around. Will you be coming here to visit me again?” A staff member is shuffling through the storage cupboard next to us, cigarette hanging out the side of his mouth for the duration. At regular intervals, he shares the cigarette, exchanging puffs with one of the female residents. It’s a ritual requiring few words.

Evening now: residents are in their rooms; individual televisions and four walls. Residents emerge occasionally to have a smoke. It provides intermittent relief in the later hours of the day and into the night. Beyond the front gate, the suburb sleeps.

(Reprinted from: Landow, J.E., Ed.; Smoking Cessation: Theory, Interventions and Prevention. New York: Nova Science Publications. Chapter One—“A Day in the Life of…”: The Culture of Cigarette Smoking for Psychiatric Populations, pp. 1–96, 2008, Lawn, S.) [29].

### 5.2. Results for the Second Study—2010

Ten years after the completion of the first study, the social milieu of the SRF settings was observed by the second researcher to be unchanged. The dominant activity in outdoor sitting areas in each SRF continued to be smoking. Following the scoping period described in the methods section in which a range of concerning anecdotal feedback about entrenched smoking issues was collected by the second researcher, they established smoking cessation support groups in each of the six SRFs that agreed to participate in the study.

The resident population at each SRF varied. One SRF housed approximately 25 residents, three SRFs each housed approximately 30 residents, and two SRFs each housed approximately 35 residents. Across the six sites, although smoking cessation support group attendance fluctuated across the sessions, a total of 277 attendees were recorded over the eight weeks. This meant that approximately 35 people participated every week across the sites. This represented approximately six participants or between 17%–24% of the total resident population at each site across the eight weeks and 18.9% of the total number of residents across all sites (*n* = 185). Information about the total number of smokers at each SRF was not collected. However, this was thought to be between 60%–80% according to SRF staff self-report. This means that almost one-third of residents who smoked at these SRFs demonstrated interest in addressing their smoking. Greater rigor in the collection of these measures was hampered by the need to accommodate residents who frequently displayed high levels of psychosocial disability. For example, some residents would come in and out of the group a number of times during the session, and some residents were unable to sit for more than 5–10 min due to their observed levels of agitation and problems with concentration.

There was a marked difference in the group program delivery format between the three areas. One northern site was fortunate to have a team of workers to facilitate the group program and a diverse range of health experts to provide participants with information about smoking cessation, including healthy behaviours and stress management. The southern sites had one primary health nurse who worked primarily one-to-one with interested residents and then referred them to the primary health care team. One location in the northern region had no workers to deliver the group program. At this site, the researcher facilitated all sessions either alone or with assistance from another Cancer Council SA worker. 

Based on self-report from residents and researcher observation, it was apparent that many SRF residents attempted quitting. At least 70 residents (37.8% of the total number of residents across the six SRFs) attended at least one of the weekly sessions and 46 residents (24.9%) were recorded as using at least one week’s supply of the free NRT products dispensed through the program. At the end of the eight-week program, a total of 17 residents (9.2%) who had been heavy smokers had quit completely, many more had substantially reduced cigarette consumption and others had tried to make changes around their smoking. Many other residents who did not smoke also participated in the sessions and reported learning skills to support others to smoke less or quit. In brief feedback to the researcher, residents and SRF staff reported that residents had enjoyed the sessions and that they had learned about the hazards of smoking and also some strategies to smoke less or quit. These group attendance numbers reflect residents only.

SRF staff could also attend the group if their work schedules permitted. Based on their interactions with SRF staff during the period, group facilitators were aware that one SRF staff member at one site had stopped smoking and others had cut down or attempted quitting. SRF staff expressed surprise at the level of dedication to quitting shown by many residents. Attitudes of staff towards residents’ smoking were observed to shift perceptibly over the eight weeks. Staff became more enthused about cessation and more supportive of organizational change efforts to address smoking in the SRF setting and the individual residents’ efforts to cut down or quit smoking (see Table 2 for more detail). Staff at each site were invited to participate in Quit SA Quitskills training in tobacco cessation. Five SRF staff from three sites completed one-day Quitskills training with a view to being a more effective support for residents to cut down or quit during and after the project.

The researcher observed that the environment at most of the six sites also changed significantly across the eight-week period. In the early stages of the project, evidence of smoking indoors was noted at some sites and no sites appeared to have a smoking policy or any procedures that attempted to manage smoking such as a designated smoking area. During the project period, the researcher perceived that the attitudes of both staff and residents had clearly shifted. Sites became visibly cleaner and enthusiasm for smoke-free practices increased as site changes and individual successes began to occur (See Box 3 Case Examples of site changes and individual success recorded by the researcher as reflective notes).

Box 3 **Case Examples of site change and individual success (2010).**

***Case Study 1: Site change***

The preliminary site visit showed that there was little control over smoking at the site apart from limiting smoking to outdoor areas. Residents were smoking at the front and back doors and near a window to the kitchen. Butts littered the ground and there was a strong smell of tobacco smoke at the premises (inside). When smoking was discussed with residents many voiced dismay at the amount of money that they “wasted” on tobacco and worry about what smoking was doing to their overall health. Staff expressed disbelief that residents would be able to make any sustained change. They felt that smoking was part of the innate nature of having a mental illness. Staff smoked with residents and residents often followed staff in the hope that they would share cigarettes or relinquish butts for residents to re-roll. Residents and staff expressed frustration at being continually hassled by some residents for tobacco products.

Once the smoking cessation support program commenced, attitudes and practices of staff and residents at the site shifted significantly. Simultaneously, the site was undergoing accreditation, the venue was being re-painted and the gardens were being replanted. As the weekly sessions commenced, people began to notice smoking, rather than it being a routine and invisible behaviour in which most at the site engaged. Management at the site decided to implement the use of a designated smoking area at the rear of the site. This was observed to de-normalize smoking and was perceived as a major factor in assisting people who were attempting to quit. It also confined litter to one spot. Passive smoking issues were vastly reduced. The site looked and smelt much more pleasant and several residents and staff commented on the new pride that they felt in their residence.

One staff member who quit smoking through the program became a “Quit Champion” and was observed on numerous occasions encouraging both residents and staff to continue trying to quit and to attend the group sessions for support. Other staff also made positive statements about the program and residents’ efforts. The ongoing impact of the program is that more residents are requesting assistance to attempt to reduce or quit smoking. Weekly, new residents are joining the program.

***Case Study 2: Individual success***

Resident J is in his 50s and had a history as a heavy smoker of tobacco (40 cigarettes per day) plus an unknown quantity of cannabis on a daily basis for many years. He has emphysema (14% lung capacity) and is very underweight. Prior to this program he had been a frequent presenter to the hospital emergency department and had multiple admissions for his physical health problems. For example, over a one-month period he was admitted to hospital five times. Following this quit program, J stopped using tobacco and cannabis. A recent one-month post review showed he called the ambulance only once during this time; however, he was not taken to hospital, as this was a panic attack. Stopping smoking has impacted upon J’s life in a number of ways. He has rejoined a choir. He has managed to save his money, which would normally have been spent on cigarettes, and spent it on purchasing a ticket for a rock concert. J is now saving with a long-term goal of going on a holiday. J has seen a dietician and is beginning to put on weight. He now has support from the respiratory integrated care services team and they are happy to have input now that J is a non-smoker. He is also socializing more.

Resident K is in his 40s and had been a smoker for many years. He has type 2 diabetes, is overweight and at risk of cardiovascular disease. In the first session he strongly defended smoking and was very verbal in his views on smoking and why he liked it. By the third session he stated that he wanted to try to stop smoking, accepting NRT lozenges to help him. By the fourth group session he had not had a cigarette and staff observed that he had begun to take more pride in his personal appearance. He became clean-shaven, had a haircut and frequently informed the group facilitator that he did not smell now. This resident explained that he had stopped going to church because smoking was against his religion and in the past people at church and those who transported him commented that he smelt of cigarette smoke. He had missed going to church but was too embarrassed to go. After quitting, he said he is now attending church again and is very happy he has returned to his church community. Resident K gave himself a weekly reward of going to the local pub to have a counter meal for dinner with the money he had saved from not smoking and also set himself a longer-term goal to save towards purchasing a car. He also began to ride his bike again and said he felt healthier. He became a support person to other residents, helping them with their attempts to quit smoking.

### 5.3. Integration of Results from the Two Study Periods

Three key interconnected themes were apparent from the results across the two study periods. The first theme was the overwhelming sense that SRFs were dispiriting environments that enhanced the rewarding value of cigarette smoking. During study 1 and study 2 prior to the group program, the researchers observed the SRFs as environments dominated by institutionalized care practices and reinforcement of a smoking culture among both staff and residents. The impoverished lifestyle and quality of life of the SRF residents was the most noticeable feature of these settings. Their day seemed to be dominated by smoking. During study 1, residents spoke and acted as if they were powerless to change any aspect of their current situation or future. Each day seemed the same, with activities and expectations having shrunk to the more immediate thoughts of wondering when and where the next cigarette was coming from. Long-term goals were spoken of as if they were dreams; the present smoking milieu totally dominating their thoughts and actions. Smoking leftover butts was common. It was more a case of who got to the ashtrays first in the early morning rather than which residents chose this level of self-degradation. Cigarettes were scarce and prized commodities.

During study 1, SRF staff was observed to supply cigarettes to residents as a routine part of the delivery of care, to reinforce cigarettes as “prized goods”. This was apparent from the daily involvement of staff in the rationing of cigarettes to more vulnerable residents. It was also apparent from multiple observations of intimidation used by some residents towards other residents to obtain cigarettes, or their attempts to trade and barter for cigarettes by offering goods such as food, soft drink, personal items, personal favours, money, and the promise of future payment. Residents were aware of staff aversion to these behaviours; however, in the absence of any meaningful action to address the underlying smoking, this awareness only served to reinforce “the game” for residents, of not getting caught by staff trading in cigarettes. Many staff were also observed to smoke with residents.

On entering the SRFs to establish smoking cessation support groups for study 2, the second researcher observed virtually the same cultural milieu. Smoking appeared to be the dominant social activity in the absence of other meaningful activities. Cigarettes were still a central part of care provision. Comments made by staff and residents to the researcher (see Table 2) during the process of scoping their interest in establishing a smoking cessation support group at their site reflected complete acceptance of smoking within the setting.

The second theme concerned the low expectations about the interest and capacity from smokers with SMI to smoke less or quit smoking. During study 1, staff demonstrated this by their complete engagement with smoking as a routine and central part of the daily life of the SRF. During study 2, both staff and residents’ comments to the researcher (see Table 2 and Case Study 1 in Box 3) upon entering the settings to establish groups demonstrated that both groups did not believe that residents could or would change their smoking behaviours. The feedback from staff, particularly their surprise about residents’ positive engagement in the group programs, demonstrated that these low expectations could be overcome. The outcomes of these group programs also challenged these assumptions for staff and residents. Almost one quarter of group participants elected to try NRT. Almost 40% of the total resident population attended at least one group session and almost 10% quit smoking. 

The third theme concerned the lack of availability or encouragement and treatment for smoking cessation for SRF residents. During study 1 and study 2 prior to commencement of the group program, there was no evidence of any encouragement or treatment being offered to support residents to cut down or quit smoking. Once the group program commenced at each site, both staff and residents’ attitudes towards smoking were observed to change. The positive impact of this relatively short period of support (eight weeks) on the overall smoking culture and structural aspects of how care was provided was evident. SRF managers instituting designated smoking areas in order to support residents who were non-smokers and those attempting to cut down or quit. Several SRF staff undertook Quitskills training. This demonstrates that they now saw it as part of their role in providing care and support to residents. As noted above, the outcomes when smoking cessation support and treatment was offered clearly demonstrate that residents did respond positively to these measures.

## 6. Discussion

Among the SRFs involved in this study, the researchers observed the presence of a strong smoking culture. This was characterized by high rates of smoking amongst residents and staff, smoking as a central mediator of many interactions between those present in the SRFs, as well as an observed lack of implementation of policy and procedures to address smoking in these SRFs. 

Results from the first study period demonstrate that these SRFs were impoverished learning environments, providing largely institutionalized care in the community and that smoking was a fundamental part of that institutionalization process. Cigarettes served many purposes within the social milieu of the SRFs. The results of the more recent study showed that many institutionalized attitudes and beliefs about smoking as inevitable for this population continue to be held by SRF staff, but that those beliefs could be challenged through the provision of tailored support to help residents to smoke less or quit. The results of the second study also show that, without assertive support to address smoking and provide smoking cessation support for SRF residents, the institutionalized pro-smoking culture would likely continue, despite many changes that have occurred in the wider community towards smoke-free policy and reduced smoking rates in the general population [2]. Across both time periods, the nature of the physical environment in which smoking interactions occurred between residents and between residents and staff was also observed to encourage or reinforce smoking and discourage quitting. In all SRFs across both time periods the main social hub of each SRF was a place where the dominant activity was smoking or the pursuit of cigarettes. Smoking cessation support therefore needs to address the impact of the environment on its members and the relationships that are fostered as a result of structures within the settings [61,62,63]. Improvements in the physical structure and appearance of the settings are indicated so that smoking is not perceived as the dominant activity. The introduction of more meaningful alternative activities is also indicated. These improvements would also ensure that SRFs were meeting the requirements of the Residential Facilities Act. In particular, providing smoking cessation support would assist in minimizing exploitation in the SRF setting.

Limited alternative activities, limited social roles, and predictable and regimented meal routines were important negative influences on the social milieu of the SRF, promoting the use of cigarettes as temporal reference points. That is, cigarette smoking routines acted as markers for orientating the residents to time, place, and person, with residents’ comments suggesting that they did not look far beyond their present situation [64]. This situation might discourage them from developing the necessary skills and motivation for long-term planning, goal-setting, and autonomous decision-making needed to consider quitting, sustain a quit attempt, and be successful at quitting smoking. The findings or this paper bolster recommendations made elsewhere [17] to introduce strategies that address these negative effects of institutionalization, relieve boredom, and foster greater participation in community activities and access to community resources and supports. These strategies may need to ‘reach in’ to this SRF population. This research showed that entering the SRF environment and offering tailored support directly within the settings could effect a positive change in attitudes and behaviours of residents and staff in the SRFs towards smoking cessation.

The system of exchange and barter of cigarettes throughout the SRF settings is an example of Skinner’s social exchange theory as an activity in subcultures [65] (see also Forsyth) [66]. Many of the interactions between residents and staff at the microsystem level mirrored this subculture in which cigarettes were a prized good. These processes were particularly evident in the first study and were still present at the beginning of the second study prior to the implementation of the group program. Staff appeared to be heavily involved in the supply of cigarettes to residents through formal and informal care arrangements. A further factor in the group dynamic is the notion of secondary adjustments that exist within institutions [67] (p. 188ff). With this comes a kind of code or informal social control. This mechanism was evident in the frequent observation of exchanges between residents that involved trade and stand-over for cigarettes. It was also evident from observation of SRF staff being fully aware of these exchanges but largely ignoring them. A shared code of conduct by residents appeared to be in operation, such as the way that they refused to tell the staff member that one of the male residents had been begging for cigarettes. It appeared to mirror the institutional social arrangements that Sykes’ described in their study of prisoners. The pecking order that Sykes described involved exchange of goods such as cigarettes, clothing, food, and gestures of deference between prisoners. Similar social exchanges were observed in the SRF sites [68]. 

Negative attitudes of SRF staff appeared to be a major barrier to achieving change in smoking behaviours in these settings. Assumptions were made that people with mental illness do not want to attempt quitting, are happy smoking, need to smoke, and have no ability to cut down or quit smoking. This situation is likely to create a self-perpetuating cycle around the hopelessness of even attempting to cut down or stop smoking, and it could undermine the self-esteem and self-efficacy of SRF residents more broadly, with potential negative impacts on other aspects of their lives [29,44,46]. This lack of belief in the ability of people with mental illness to quit smoking has been reported widely in the literature [30,69,70,71].

Results from the second study confirm that many residents in these SRFs were interested in and able to cut down or quit smoking with increased support, but that a smoking culture would likely persist without such support. Other researchers have found that people with SMI are just as motived to quit smoking as those without mental illness [38,72]. The study showed that a group framework and education tailored to the needs of SRF residents, dedicated personnel to run smoking cessation support groups and financial support to fund NRT for residents are likely needed to support success of their smoking cessation efforts. Smoking cessation group programs have been shown to be successful for people with mental illness in both community and inpatient settings [38,73,74,75]. SRF residents should not be excluded from such programs. However, structural changes to the SRF setting in parallel with group program attendance or individual smoking cessation support is needed to ensure that any gains made by residents are not undermined by an overwhelming environment that reinforces and rewards smoking. The results of the current study suggest that some SRF residents can and do quit smoking and may sustain non-smoking practices when offered appropriate support. 

The results of the two studies reported here suggest that a comprehensive and systematic approach to addressing smoking among SRF residents (and staff) is needed. SRF residents are amongst the most vulnerable in the community. Many SRF residents live with multiple psychosocial disadvantages and this situation is likely compounded by the cost of tobacco products in the context of 85% of their total pension income assigned to SRF boarding fees [13,14]. Changing the SRF environment from one that normalizes smoking to one that supports smoking cessation and smoke-free policy will need strong and effective advocacy from the organizations and individuals that provide care to SRF residents. 

The 2010 study reported here involved only 10% of the total number of South Australia’s SRFs. In order to achieve system-wide improvement in smoking rates for SRF populations, government guidelines must reflect the imperative to provide a clean, safe and smoke-free environment for residents and staff in SRFs. This can only be achieved if owners and managers of SRFs also show a commitment to tackling tobacco through systematic protocols that are uniform across the sector. SRF staff commitment and training is needed to assist SRF residents attempting to smoke less or quit. This system-wide approach will ensure that SRF residents will receive consistent smoking cessation support regardless of the SRF in which they reside. Messages that support quitting smoking also need to be clear and delivered in a variety of ways, utilizing different media and formats to accommodate SRF residents’ varying levels of literacy and psychosocial disability. More visual and sensory resources may be required for this setting. [76]. A systems approach could include, but not be limited to the strategies outlined in Box 4.

Box 4 **Strategies to support smoking cessation in supported residential facilities.**

SRF staff could ask and record smoking status as a standard component of collecting the personal data of each SRF resident.

Upon entry into the SRF system, assistance to quit smoking could be offered to all residents who smoke. This assistance could continue on a regular basis.

SRF staff job advertisements could mention the smoking cessation support initiatives and smoke-free status of the SRF facilities. The position descriptions for prospective employees could require them to be non-smokers or ask about their willingness and ability to comply with smoke free policies and procedures.

SRF staff who smoke could be offered assistance to quit smoking as a standard component of staff care and support to perform their duties.

Policies and procedures could include a staff code of behaviour that requires them to not smoke with residents whilst on duty. Friends, family, and others who are visiting the SRF could also be bound by these rules while they are at the site.

SRFs could confine on-site smoking by residents to a “designated smoking area” located well away from thoroughfares, doorways, open windows, and air-intake points. This area could be designed so that it does not facilitate peer socializing while smoking.

SRF staff could be provided with routine training in smoking cessation support. This could include information about how to use smoking cessation pharmacotherapy and how to make referrals to appropriate supports such as the Quitline or a General Practitioner.

SRF staff could offer residents a variety of appropriate alternative activities to alleviate boredom and stress. Community welfare organizations could provide greater reach into the SRFs to provide social and recreational activities to SRF residents. Where appropriate, residents could be encouraged and supported to engage with the wider community and participate in meaningful community-based activities and events.

Friends and family of residents could be encouraged to no longer supply residents with tobacco products, smoke at the site, or bring tobacco products to the site.

## 7. Limitations

There are several limitations of this research that indicate that a larger, more systematic approach to studying the smoking cessation support needs of SRF residents is needed. These two studies were conducted in Adelaide, Australia and results may reflect policy and practice in that jurisdiction with limited generalizability to SRF environments in other countries. Housing policy, smoke-free policy, funding, connection to mental health services, make up of resident population, and many other variables may vary depending on the nature of arrangements for this population group in different countries. 

In the first study, the validity and genuineness of observations was enhanced by habituation of the settings to lessen the researcher’s obtrusiveness in the settings and to promote familiarity with participants and their routines. As well as this, the researcher did not make it known to SRF staff or residents exactly when she was recording data and when she was merely observing. This overcame “staged” interactions for the researcher’s benefit. Therefore, observations made were more likely to be valid and reliable than if this precaution had not been taken. The first author undertook all observations at the SRF sites; therefore, there is potential for observer drift [77]. One solution would be to have more than one researcher performing this role and to conduct inter-observer agreement checks to minimize this potential problem [77]. To combat this potential problem, the researcher trialed observation sheets and regularly debriefed with supervisors. She also kept copious reflective, descriptive notes about the observations. She was disciplined in recording these notes as soon after the observations as practicable. The use of standardized observation sheets, based on the results of the fully audited qualitative data prior to entering the field also helped to overcome potential threats to validity and reliability and to enhance replicability.

There is also scope for improving the process of recording observations. In this study, observations were limited to one area of each SRF and therefore did not capture smoking that may have been occurring simultaneously in other locations at the sites, such as inside and at the front of the SRF. Also, the counting methods used did not account for the fact that, if a smoker entered the smoking area more than once during the three-hour period of observation, they were counted each time they re-entered. This hampered the researcher’s ability to determine smoking activities by people in the observed areas as a percentage of overall number of residents of the SRFs. 

The second study has several limitations related to its design, in particular, the rigor of data collection, analysis, and reporting of the efficacy of the interventions used in this study. Most residents and SRFs opted in, were not blinded to changes in smoking policy and the intervention, were not randomized to treatment conditions, and there was no parallel control group to compare effects and outcomes. A more systematic and rigorous design is needed as part of a larger, adequately powered randomized controlled trial. Despite this limitation, multiple examples of positive changes in smoking behaviour were observed following the smoking cessation group intervention. Another limitation is that, although each group facilitator was provided with QuitSkills training, there may have been variation in how smoking cessation groups were delivered at each site. Other variables that may have impacted on residents’ engagement with the groups were not collected; for example, there is no detailed information about their diagnoses, other psychosocial issues, level of nicotine dependence, past quit attempts, or levels of social support available to them. The absence of in-depth interviews with SRF residents and staff, or other means of gathering a detailed account of their perspectives, was a significant limitation of this study. A further limitation of the second study was that the SRFs and their residents self-selected to be involved in the study. This suggests that these sites were more progressive and that their residents and staff were more motivated to address smoking. However, it may be that attitudes and smoking environments in those SRFs that were not included in the study are even more defeatist and conducive to smoking than those captured at baseline for the included sites. 

## 8. Conclusions

Despite growing initiatives in the wider community to address the high rates of smoking by people with mental illness, a powerful and pervasive pro-smoking culture has continued within the SRFs involved in this study. This is characterized by high rates of smoking observed among residents and staff, as well as an obvious lack of consistent policy and procedures to address smoking behaviours in these SRFs. There appears to be energy and expertise available from health and community service providers and from within the SRF sector in South Australia to address smoking and improve the conditions that underpin the health and wellbeing of residents of SRFs. Many SRF residents also appear to want to smoke less or quit when given adequate support. However, there are a range of system factors such as low expectations about the interest and capacity of smokers with SMI to quit, SRF environments that enhance the rewarding value of cigarette smoking, and a general lack of encouragement and treatment for smoking cessation for SRF residents that undermine these efforts. This pro-smoking culture within the SRF sector will continue to undermine these efforts unless upstream prevention and early intervention strategies are also implemented. A systems approach is required to change entrenched attitudes and practices and to truly embed sustainable change in the SRF sector. Widespread cultural and organizational change in the sector is unlikely without the adoption of service-wide uniform protocols that inform, educate, and encourage managers, staff, and individual residents to embrace smoking cessation support and smoke-free practices. Some progress has been made; however, this appears to be piecemeal. Ideally, this systems approach should be linked to any State or Federal Government funding and accreditation for SRF sites to ensure best practice around tobacco control and that smoking cessation support is implemented and sustained. 

## Figures and Tables

**Table 1:** Summary of observations from the supported residential facilities’ smoking areas.

Observations	SRF 1 (*n* = 24 Residents)	SRF 2 (*n* = 24 Residents)	Combined Total
Broad Descriptive Information About Observations			
Number of visits to the SRF	6	6	12
Total number of observation hours	24	24	48
Number of observation hours (sequential and frequency data)	18	18	36
Detailed Frequency Information About Observations *			
Total number of people in smoking area	371	530	901
Number of smokers	332 (89.4%)	475 (89.6%)	799 (89.5%)
Number of cigarettes consumed	361	517	878
Number of SRF residents in smoking area	363 (97.8%)	499 (94.2%)	862 (96%)
Number of SRF staff in smoking area	8 (2.2%)	31 (5.8%)	39 (4.3%)
Number of passive smokers in smoking area **^#^**	49 (13.2%)	55 (10.4%)	104 (11.5%)

* Observation sheets for collecting data about these variables used 10-min intervals as the recording parameters because this denoted the average time taken to smoke a cigarette. Each person needed to be in the observed area for at least 5 min in the 10-min interval in order to be counted; **^#^** To be counted as a passive smoker, the person was required to be present in the observed area for 5 min or more.

**Table 2:** Comparison of SRF staff comments.

SRF Staff Comments Prior to Commencement of the Program	Staff Comments during and after the Program
“Mental health and smoking go together don’t they?”. “You won’t be able to change anyone”. “They have always smoked—it’s what gets them through the day isn’t it?”. “They have all had hard lives, smoking is all they have”. “They enjoy it they do not have much in their lives—why try and take the one thing they enjoy away from them”. “Smoking is a comfort for them, they’re happy when they sit together and smoke”. “Even if they try to stop they will give up they won’t be able to do it”. “They will never do it”. “They (residents) always smoke out the front; you won’t stop them—we have tried and they still do it”.	“I am shocked that they do not smoke in the front (yard) anymore”. “The front entrance looks so clean we have planted some new plants where the old ones kept dying”. ”We haven’t got all those horrible butts to clean up”. “I didn’t believe (Mr X) would be able to make any change, and look at how well he has done. He is much healthier. Previously he would have been rushed to hospital on a regular basis. Since the program, he hasn’t been unwell once. It’s amazing”. “I am shocked that they (residents) sat and listened”. “I am really surprised at the amount of knowledge that residents had about smoking”. “I never thought that he would even think about not smoking”. “She has still been smoking but she has cut back a lot and that is massive for her”. “It is so hard for him but he is doing so well—we are trying to support him”.

## Appendix: Smoking Area Observation Sheets

**Sheets A1:** General frequency observation variable.

Obs. Freq. Measures	Ten Minute Time Intervals																	
	10	20	30	40	50	60	70	80	90	100	110	120	130	140	150	160	170	180
(1) Number of Cigarettes Consumed																		
(2) Number of Smokers																		
(3) Number of Group																		
- Residents																		
- Staff																		
(4) Number of Passive Smokers																		
**Time of Day From:** **To:**																		

**Sheets A2:** Qualitative description of the setting.

**Location:**	
	
**People Present:**	
	
	
	
	
**Physical Description:**	
	
	
	
	
	
	
**Activities:**	
**(and absence of)**	
	
	
**Time of Day:**	
	

**Sheets A3:** Sequences of smoking area interaction—residents and staff.

**Activity: (person 1)**									
- smoking									
- non-smoking									
**Other Activity: (person 1)**									
- talking									
- sitting silently									
- pacing									
- coughing									
**Proximity:**									
- together/socialising									
- separate									
- nearby/not socialising									
- intrusive									
- alone									
**Enter-Staff: (person 2)**									
- joins									
- offers smoke/light (1 > 2)									
- offers smoke/light (2 > 1)									
- leaves (person 1)									
- 1 ignores 2									
- 2 ignores 1									
- 1 moves away									
- 1 continues activity									
- 1 stops activity									
**Enter-Client (person 3)**									
- joins									
- remains separate									
- offers smoke/light (1 > 2)									
- offers smoke/light (2 > 1)									
- leaves (person 1)									
- 1 ignores 2									
- 2 ignores 1									
- 1 moves away									
- 1 continues activity									
- 1 stops activity									
- asks for smoke/given									
- asks for smoke/denied									
- intimidate for smoke (1 > 2)									
- intimidate for smoke (2 > 1)									

## References

[1] Cooper J., Mancuso S.G., Borland R., Slade T., Galletly C., Castle D. (2012). Tobacco smoking among people living with a psychotic illness: The second australian survey of psychosis. Aust. N. Z. J. Psychiatry.

[2] Scollo M.M., Winstanley M.H. (2015). Tobacco in Australia: Facts and Issues.

[3] Morgan V.A., Waterreus A., Jablensky A., Mackinnon A., McGrath J.J., Carr V., Bush R., Castle D., Cohen M., Harvey C. (2012). People living with psychotic illness in 2010: The second Australian national survey of psychosis. Aust. N. Z. J. Psychiatty.

[4] Gfroerer J., Dube S.R., King B.A., Garrett B.E., Babb S., McAfee T. (2013). Vital signs: Current cigarette smoking among adults aged greater than or equal to 18 years with mental illness—United States, 2009–2011. MMWR.

[5] Gelenberg A.J., Jose de Leon A., Parks J.J., Rigotti N.A. (2008). Smoking cessation in patients with psychiatric disorders. J. Clin. Psychiatry.

[6] Prochaska J.J. (2009). Ten critical reasons for treating tobacco dependence in inpatient psychiatry. J. Am. Psychiatr. Nurses Assoc..

[7] Access Economics (2007). Smoking and Mental Illness: Costs. Report for Sane Australia.

[8] Prochaska J.J. (2011). Smoking and mental illness—Breaking the link. N. Engl. J. Med..

[9] Prochaska J.J., Hall S.E., Delucchi K., Hall S.M. (2013). Efficacy of initiating tobacco dependence treatment in inpatient psychiatry: A randomized controlled trial. Am. J. Public Health.

[10] Stockings E., Bowman J., McElwaine K., Baker A., Terry M., Clancy R., Bartlem K., Wye P., Bridge P., Knight J. (2013). Readiness to quit smoking and quit attempts among australian mental health inpatients. Nicotine Tob. Res..

[11] Lawrence D., Lawn S., Kisely S., Bates A., Mitrou F., Zubrick S.R. (2011). The potential impact of smoke-free facilities on smoking cessation in people with mental illness. Aust. Z. J. Psychiatry.

[12] Cleary M., Woolford P., Meehan T. (1998). Boarding house life for people with mental health illness: An exploratory study. Aust. N. Z. J. Ment. Health Nurs..

[13] Doyle M., Hume A., McAvaney J., Rogers N., Stephenson T. (2003). Somewhere to call home: Supported residential facilities: The sector, its clientele and its future. Government Printer, Adelaide.

[14] Government of South Australia Department of Families and Social Inclusion (2013). Supported Residential Facilities Advisory Committee Annual Report 2011–2012.

[15] Hinton T., Evans N., Jacobs K. (2001). Healthy Hostels: A Guide to Promoting Health and Well-Being among Homeless People.

[16] Power J., Mallat C., Bonevski B., Nielssen O. (2015). An audit of assessment and outcome of intervention at a quit smoking clinic in a homeless hostel. Australa Psychiatry.

[17] Morgan D.G., Stewart N.J. (1998). High versus low density special care units: Impact on the behaviour of elderly residents with dementia. CJA/RCV.

[18] Skea D., Lindesay J. (1996). An evaluation of two models of long-term residential care for elderly people with dementia. Int. J. Geriat. Psychiatry.

[19] Te Boekhorst S., Depla M.F., de Lange J., Pot A.M., Eefsting J.A. (2009). The effects of group living homes on older people with dementia: A comparison with traditional nursing home care. Int. J. Geriat. Psychiatry.

[20] Joseph A. (2006). Health Promotion by Design in Long-Term Care Settings.

[21] Chilvers R., Macdonald G., Hayes A. (2006). Supported housing for people with severe mental disorders. Syst. Rev..

[22] (2016). Supported Residential Facilities Act 1992.

[23] Australian Bureau of Statistics (2003). 3222.0—Population Projections, Australia, 2002 to 2101.

[24] Australian Bureau of Statistics (2012). 1367.0—State and Territory Statistical Indicators, 2012.

[25] Taylor T.L., Killaspy H., Wright C., Turton P., White S., Kallert T.W., Schuster M., Cervilla J.A., Brangier P., Raboch J. (2009). A systematic review of the international published literature relating to quality of institutional care for people with longer term mental health problems. BMC Psychiatry.

[26] Baker F., Douglas C. (1990). Housing environments and community adjustment of severely mentally ill persons. Community Ment. Health J..

[27] Horan M.E., Muller J.J., Winocur S., Barling N. (2001). Quality of life in boarding houses and hostels: A residents’ perspective. Community Ment. Health J..

[28] Fakhoury W.K., Murray A., Shepherd G., Priebe S. (2002). Research in supported housing. Soc. Psychiatry Psychiatr. Epidemiol..

[29] Lawn S., Landow J.E. (2008). A day in the life of....: The culture of cigarette smoking for psychiatric populations. Smoking Cessation: Theory, Interventions and Prevention.

[30] Lawn S.J. (2004). Systemic barriers to quitting smoking among institutionalised public mental health service populations: A comparison of two Australian sites. Int. J. Soc. Psychiatry.

[31] Connor E.S., Cook L.R., Herbert I.M., Neal M.S., Williams T.J. (2002). Smoking cessation in a homeless population: There is a will, but is there a way. J. Gen. Int. Med..

[32] Goldade K., Whembolua G.-L., Thomas J., Eischen S., Guo H., Connett J., Des Jarlais D., Resnicow K., Gelberg L., Owen G. (2011). Designing a smoking cessation intervention for the unique needs of homeless persons: A community-based randomized clinical trial. Clin. Trials.

[33] Ojo-Fati O., John F., Thomas J., Joseph A.M., Raymond N.C., Cooney N.L., Pratt R., Rogers C.R., Everson-Rose S.A., Luo X. (2015). Integrating smoking cessation and alcohol use treatment in homeless populations: Study protocol for a randomized controlled trial. Trials.

[34] Okuyemi K.S., Thomas J.L., Hall S., Nollen N.L., Richter K.P., Jeffries S.K., Caldwell A.R., Ahluwalia J.S. (2006). Smoking cessation in homeless populations: A pilot clinical trial. Nicotine Tob. Res..

[35] Shelley D., Cantrell J., Wong S., Warn D. (2010). Smoking cessation among sheltered homeless: A pilot. Am. J. Health Behav..

[36] Torchalla I., Strehlau V., Okoli C.T., Li K., Schuetz C., Krausz M. (2011). Smoking and predictors of nicotine dependence in a homeless population. Nicotine Tob. Res..

[37] Chen J.S., Nguyen A.H., Malesker M.A., Morrow L.E. (2016). High-risk smoking behaviours and barriers to smoking cessation among homeless individuals. Respir. Care.

[38] Ashton M., Miller C.L., Bowden J.A., Bertossa S. (2010). People with mental illness can tackle tobacco. Aust. N. Z. J. Psychiatry.

[39] Banham L., Gilbody S. (2010). Smoking cessation in severe mental illness: What works?. Addiction.

[40] Ashton M., Rigby A., Galletly C. (2015). Evaluation of a community-based smoking cessation programme for people with severe mental illness. Tob. Control.

[41] Burdekin B. (1993). National Inquiry into the Human Rights of People with Mental Illness.

[42] Mental Health Council of Australia (2005). Not for Service: Experiences of Injustice and Despair in Mental Health Services in Australia.

[43] National Mental Health Commission (2015). Contributing Lives, Thriving Communities—Review of Mental Health Programmes and Services.

[44] Lawn S. (2001). Systemic Barriers to Quitting Smoking among Institutionalised Public Mental Health Service Populations. Ph.D. Thesis.

[45] Lucas T. (2011). Tackling Tobacco in Supported Residential Facilities Pilot Program. Final Unpublished Report.

[46] Lawn S.J., Pols R.G., Barber J.G. (2002). Smoking and quitting: A qualitative study with community-living psychiatric clients. Soc. Sci. Med..

[47] Friedrichs J., Lüdtke H. (1975). Participant Observation: Theory and Practice.

[48] Jorgensen D.L. (1989). Participant Observation: A Methodology for Human Studies.

[49] Patton M.Q. (1980). Qualitative Evaluation Methods.

[50] Simon A., Boyer E.G. (1974). Mirrors for Behaviour III. An Anthology of Observation Instruments.

[51] National Ethics Advisory Committee (2012). Ethical Guidelines for Observational Studies: Observational Research, Audits and Related Activities, Revised Edition.

[52] National Health and Medical Research Council (NHMRC) (2015). National Statement on Ethical Conduct in Human Research (2007)—Updated May 2015.

[53] Emden C. (1998). Conducting a narrative anlysis. Collegian.

[54] Kelly T., Howie L. (2007). Working with stories in nursing research: Procedures used in narrative analysis. Int. J. Ment. Health Nurs..

[55] Quit SA & Smoking and Mental Illness Project (2004). I’m Giving It a Go: A Step by Step Guide for People with Mental Illness Who Smoke and Want to Cut Down or Quit.

[56] Heatherton T.F., Kozlowski L.T., Frecker R.C., Fagerstrom K.O. (1991). The Fagerstrom test for nicotine dependence: A revision of the Fagerstrom Tolerance Questionnaire. Br. J. Addict..

[57] Guba E.G. (1981). Criteria for assessing the trustworthiness of naturalistic inquiries. ECTJ.

[58] Rodgers B.L., Cowles K.V. (1993). The qualitative research audit trail: A complex collection of documentation. Res. Nurs. Health.

[59] Rodwell M.K., Byers K.V. (1997). Auditing constructivist inquiry: Perspectives of two stakeholders. Qual. Inq..

[60] Rose K., Webb C. (1998). Analyzing data: Maintaining rigor in a qualitative study. Qual. Health Res..

[61] Moos R.H., Insel P.M. (1974). Issues in Social Ecology: Human Milieus.

[62] Moos R.H. (1976). The Human Context: Environmental Determinants of Behaviour.

[63] Blair D.T., New S.A. (1991). Assaultive behaviour: Know the risks. J. Psychosoc. Nurs. Ment. Health.

[64] Suto M., Frank G. (1994). Future time perspective and daily occupations of persons with chronic schizophrenia in a board and care home. Am. J. Occup. Ther..

[65] Skinner B.F. (1953). Science and Human Behaviour.

[66] Forsyth D.R. (1999). Group Dynamics.

[67] Goffman E. (1961). Asylums: Essays on the Social Situation of Mental Patients and Other Inmates.

[68] Sykes G.M. (1958). The Society of Captives: A Study of a Maximum Security Prison.

[69] Allan J. (2013). Smoking: Time for the mental health system to confront its own ambivalence. Australas Psychiatry.

[70] Royal College of Physicians and Royal College of Psychiatrists (2013). Smoking and Mental Health.

[71] Wye P., Bowman J., Wiggers J., Baker A., Carr V., Terry M., Knight J., Clancy R. (2010). Providing nicotine dependence treatment to psychiatric inpatients: The views of Australian nurse managers. J. Psychiatr. Ment. Health Nurs..

[72] Moeller-Saxone K. (2008). Cigarette smoking and interest in quitting among consumers at a psychiatric disability rehabilitation and support service in victoria. Aust. N. Z. J. Public Health.

[73] Barnett P.G., Wong W., Jeffers A., Hall S.M., Prochaska J.J. (2015). Cost-effectiveness of smoking cessation treatment initiated during psychiatric hospitalization: Analysis from a randomized, controlled trial. J. Clin. Psychiatry.

[74] Bonevski B., Paul C., D’Este C., Sanson-Fisher R., West R., Girgis A., Siahpush M., Carter R. (2011). RCT of a client-centred, caseworker-delivered smoking cessation intervention for a socially disadvantaged population. BMC Public Health.

[75] Williams J.M., Anthenelli R.M., Morris C.D., Treadow J., Thompson J.R., Yunis C., George T.P. (2012). A randomized, double-blind, placebo-controlled study evaluating the safety and efficacy of varenicline for smoking cessation in patients with schizophrenia or schizoaffective disorder. J. Clin. Psychiatry.

[76] Cancer Council NSW (2008). Addressing Smoking in Community Service Organisations: A Policy Toolkit.

[77] Robson C. (1993). Real World Research: A Resource for Social Scientists and Practitioner Researchers.

